# Sympathetic signals beneath the surface: fresh insights from skin and muscle

**DOI:** 10.3389/fnins.2025.1617735

**Published:** 2026-01-29

**Authors:** Tina Tian, Patricia Jillian Ward

**Affiliations:** Department of Cell Biology, School of Medicine, Emory University, Atlanta, GA, United States

**Keywords:** beta2 adrenergic receptor, neuromuscular junction, surgical reconstruction, sympathectomy, sympathetic nervous system, sympathetic skin response (SSR)

## Abstract

The sympathetic branch of the autonomic nervous system, known for its governance of the “fight or flight” response, has attracted newfound interest due to its role in maintaining bodily homeostasis in various tissue types. Sympathetic activity in the skin is often perturbed in neurological and neurodegenerative disorders. Notably, aberrant changes in the sympathetic skin response can be detected before clinical manifestations of diabetic neuropathy. Furthermore, sympathetic signaling at neuromuscular junctions in skeletal muscle has now been demonstrated to be critical for synapse integrity and proper functioning. Insufficient sympathetic signaling in skeletal muscle underlies the pathogenesis of muscle weakness in several disease states, such as myasthenia syndromes and sarcopenia. Additionally, surgical sympathectomies, a treatment method for conditions that involve heightened sympathetic activity, can give rise to other unwanted side effects, prompting the need for sympathetic trunk reconstruction. Therefore, the sympathetic nervous system, with renewed appreciation of its known functions and developing excitement for its recently discovered functions, remains a source for a wealth of potential discoveries that can further enable us to improve human health.

## Introduction

The sympathetic nervous system is a component of the autonomic nervous system that is critical for the maintenance of bodily homeostasis. Classically, the sympathetic nervous system governs the “fight or flight” response, which consists of pupillary dilation, sweating, increased respiratory rate and heart rate, muscle tension, amongst other physical manifestations (McCorry, 2007; Dibona, 2002; Cabezas et al., 1971; Lowenstein and Loewenfeld, 1950; Uno, 1977; Davis et al., 1977; Grassi et al., 1998). Outside of the functions classically associated with the “fight or flight” response, the sympathetic nervous system also regulates the immune system, gluconeogenesis, bone remodeling, circadian rhythm, fat metabolism, and skeletal muscle health and function (McCorry, 2007; Besedovsky et al., 1979; Carnagarin et al., 2018; Dibona, 2002; Elefteriou et al., 2014; Elenkov et al., 2000; François et al., 2019; Khan et al., 2016; Lee et al., 2010; Rodrigues et al., 2019). The sympathetic nervous system is an antithesis to the “rest and digest” response of the parasympathetic nervous system, and these two components of the autonomic nervous system work together to maintain homeostasis.

This review emphasizes the rising appreciation of the breadth of functions that the sympathetic nervous system governs in peripheral tissues, namely the skin and muscle. The clinical relevance of sympathetic activity in skin and muscle is notable in many disease states, and there has been renewed interest and evidence supporting sympathetic signaling at the neuromuscular junction (NMJ), that the effects of sympathetic muscle innervation go further than vascular regulation. Although traditionally described as independent from one another (Delius et al., 1972; Derius, 1972; Hagbarth et al., 1972), sympathetic activity in skin and muscle are related in certain neurological disorders involving autonomic dysfunction. For example, amyotrophic lateral sclerosis (ALS), the hallmark symptom of which is primarily muscle weakness, can present with hyperhidrosis as an initial symptom (Giugno et al., 2022; Chen et al., 2024). In advanced stages of ALS, there is subsequent decline in sweating, likely indicative of progressive deterioration of the sympathetic nervous system (Beck et al., 2002). Finally, recent efforts to surgically reconstruct the sympathetic chain ganglia after sympathectomies in patients underscore the importance of an intact sympathetic nervous system (Chang et al., 2021; Chen et al., 2023).

## Sympathetic activity in skin

**Figure 1:**
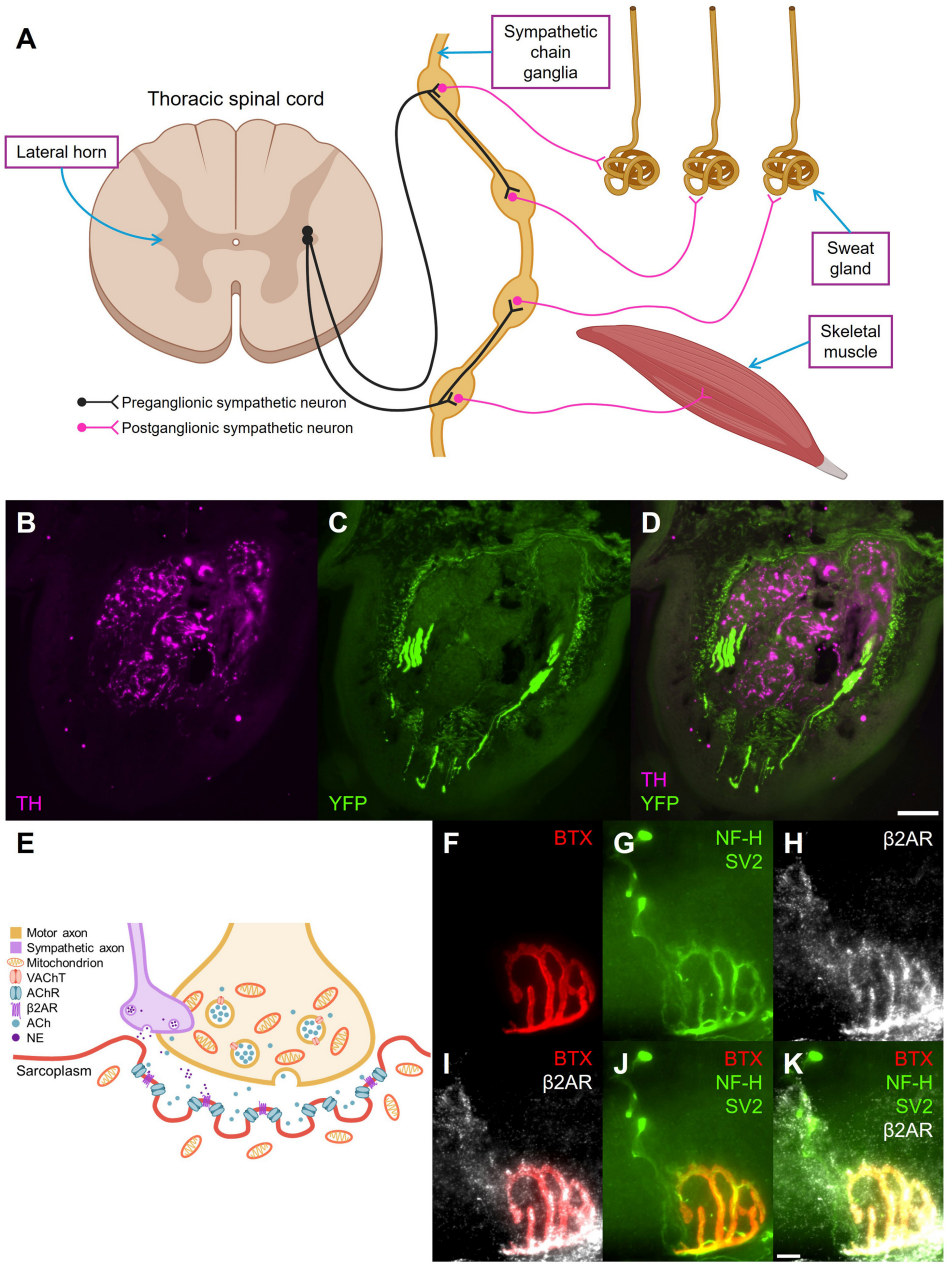
Anatomical overview of the sympathetic innervation of skin and muscle. **(A)** Two-neuron pathway of sympathetic innervation of sweat glands and skeletal muscle. **(B)** Sympathetic fibers in sweat glands (TH, magenta), **(C)** sensory fibers in skin (YFP, green), avoiding the sweat gland area, and **(D)** merged sympathetic and sensory fibers in a cross-section of a footpad of a YFP-16 mouse, a reporter mouse with negligible YFP expression in postganglionic sympathetic neurons (Feng et al., 2000). TH, tyrosine hydroxylase; YFP, yellow fluorescent protein. Scale bar = 100 μm. **(E)** Schematic diagram of sympathetic contribution to neuromuscular junctions (NMJs). **(F)** Postsynaptic acetylcholine receptors (BTX, red), **(G)** motor fiber (NF-H, SV2, green), **(H)** postsynaptic β2-adrenergic receptors (β2AR, white), **(I)** overlap of motor and sympathetic receptors, **(J)** overlap of motor fiber and motor receptors, **(K)** and all merged in a mouse gastrocnemius NMJ. VACHT, vesicular acetylcholine transporter; AChR, acetylcholine receptor; β2AR, β2 adrenergic receptor; ACh, acetylcholine; NE, norepinephrine; BTX, α-bungarotoxin; NF-H, neurofilament-heavy chain; SV2, synaptic vesicle glycoprotein 2. Scale bar = 10 μm.

Both glabrous and hairy skin are abundantly innervated by the sympathetic nervous system, with cutaneous effectors making up 25%−50% of neurons in the sympathetic chain ganglia (Donadio et al., 2006; Gibbins, 1997). The manifestations of sympathetic activity in skin include sweating, vasoconstriction and vasodilation, and piloerection (Everett IV et al., 2017). The majority of sympathetic skin innervation is norepinephrine-mediated, also known as noradrenergic fibers, which regulate vasoconstriction and piloerection (Gibbins, 1997), while a subset of cholinergic sympathetic fibers is concentrated in sweat glands and ducts (Figure 1A; Schütz et al., 1998). In glabrous skin, sympathetic noradrenergic fibers are primarily located around arteriovenous anastomoses (Donadio et al., 2006), which are thermoregulatory structures (Morris, 1997), while noradrenergic fibers in hairy skin are primarily present in arrector pili muscles (Donadio et al., 2006).

Eccrine sweat glands are thermoregulatory structures that are present all over the body, but are especially concentrated on the palms of the hands and soles of the feet, and are important effectors of the central autonomic network during homeostatic adjustments to body temperature and responses to emotional and gustatory stimuli (Vetrugno et al., 2003; Groscurth, 2002; Kreyden and Scheidegger, 2004; Benarroch, 1993). Human sweat glands are innervated by postganglionic sympathetic axons that are primarily cholinergic (Kahn and Rothman, 1942). (Donadio et al., 2006; Collins, 1989). The anatomical distribution of eccrine sweat glands in other primates mirrors that seen in humans (Johnson and Elizondo, 1974). In mice, eccrine sweat glands are only found on the footpads and resemble human eccrine sweat glands when they are mature (Taylor et al., 2012). This distribution found in mice is recapitulated in other mammals, such as cats, rats, and hedgehogs (Taylor et al., 2012; Stumpf et al., 2004). The limited distribution of eccrine sweat glands in these animals suggests that sweating plays more of a role in increasing foot traction for mobility and tactile sensation of the foot rather than for thermoregulation (Stumpf et al., 2004; Taylor et al., 2012). Mouse sweat glands exhibit robust tyrosine hydroxylase immunoreactivity, which is indicative of their sympathetic innervation (Rao et al., 1994; Figures 1B–D).

The **sympathetic skin response (SSR)** is the voltage change of the skin surface in response to various stimuli, most commonly electrical stimulation of the peripheral nerve, due to activation of a polysynaptic reflex arch (Kucera et al., 2004). The SSR can be used as a noninvasive test to study sympathetic function (Arunodaya and Taly, 1995). The voltage change is activated by cholinergic fibers that innervate eccrine sweat glands. The SSR reliably detects disorders that affect distal sympathetic axons but does not correlate well with clinical dysautonomia symptoms (Shahani et al., 1984; Kucera et al., 2004). Since the SSR only specifically tests the functional sympathetic innervation in the skin, the vast functional repertoire of the autonomic nervous system is impossible to encompass with just the SSR. Therefore, the SSR is better at documenting axonal pathology, such as that found in peripheral neuropathies, in which the distal sympathetic fibers innervating the skin “die back” long before other more central autonomic fibers are affected to cause orthostatic hypotension and other functional signs of dysautonomia (Goadby and Downman, 1973; Fagius and Wallin, 1980).

Clinically, the SSR is used in the diagnostic workup of patients with suspected autonomic disorders in the setting of peripheral neuropathy, most frequently diabetic peripheral neuropathy (Kucera et al., 2004; Niakan and Harati, 1988; Soliven et al., 1987; Braune and Horter, 1996). However, abnormal SSR is also detected in many other neuropathies ranging from hereditary to alcoholic to inflammatory conditions, and disease severity is often correlated with worsening of the SSR (Niakan and Harati, 1988; Braune and Horter, 1996). Even in early-stage diabetic neuropathy, the majority of patients exhibit abnormal SSR changes (Braune and Horter, 1996). Therefore, SSR testing can be used to assess autonomic disturbances even in diabetic patients who show little to no clinical signs of peripheral neuropathy (Niakan and Harati, 1988; Braune and Horter, 1996). Regarding central nervous system disorders, SSR abnormalities are detected in the majority of patients with multiple sclerosis and are associated with disease severity (quantified by total lesion volume) and the locations of certain lesions (Yokota et al., 1991; Gutrecht et al., 1993; Saari et al., 2008).

People with Parkinson's disease (Hirashima et al., 1996; Schestatsky et al., 2006), ALS (Dettmers et al., 1993; Hu et al., 2016), syringomyelia (Veciana et al., 2007), Huntington's disease (Sharma et al., 1999; Andrich et al., 2002), and more also exhibit pathological changes in the SSR. These neurological disorders also have hallmark motor neuron symptoms, namely ALS, which is often diagnosed due to progressive muscle weakness as the presenting symptom and is characterized as a motor neuron disease (Zoccolella et al., 2006). Even so, the average time from the first symptom to diagnosis is 14.4 months. Autonomic impairment has been frequently described in ALS with symptoms that span from cardiovascular to urinary to thermoregulatory to gastrointestinal (Piccione et al., 2015; Sachs et al., 1985; Chida et al., 1989; Dettmers et al., 1993; Toepfer et al., 2000, Beck et al., 2002; Baltadzhieva et al., 2005; Zoccolella et al., 2006; Hu et al., 2016). Notably, there has been a recent resurgence of interest in autonomic dysfunction in ALS (Chen et al., 2024; Dubbioso et al., 2023; Oprisan and Popescu, 2023; Papadopoulou et al., 2022), with some evidence to suggest that sympathetic impairment in the form of hyperhidrosis could be an early symptom before motor neuron symptoms begin to manifest (Chen et al., 2024). Furthermore, patients with early stages of ALS sweat more than control subjects, and as the disease advances, the sweating response is decreased compared to controls, suggesting progressive degeneration of postganglionic sympathetic axons (Beck et al., 2002; Mazzaro et al., 2023).

Overall, the sympathetic innervation of skin is abundant and primarily plays roles related to thermoregulation. Abnormalities in sympathetic signaling lead to changes in the SSR, which is defined by a voltage change in the skin that is evoked by the effector cholinergic fibers that innervate eccrine sweat glands. These abnormalities in SSR are associated with many different types of peripheral neuropathy as well as autoimmune diseases and neurodegenerative disorders. These findings drive home the potential diagnostic utility of sympathetic skin activity, illustrating the pervasiveness of autonomic dysfunction in neurological disorders, even in early and subclinical stages of disease.

## Sympathetic signaling in skeletal muscle: more than just vessels

The presence of sympathetic signaling in skeletal muscle is not a novel concept, with reports of sympathetic innervation to skeletal muscle dating back to the 1910s and 1920s with the first report of unmyelinated nerve fibers in skeletal muscle being published in 1879 (Tower, 1926; Forbes, 1929, Kuntz and Kerper, 1926, 1924; Hunter, 1924; Kuno, 1915; Agduhr, 1920; Coates and Tiegs, 1928). These unmyelinated fibers were first proposed to be sympathetic fibers (Figure 1A), and important for skeletal muscle tone in 1904 by Perroncito and Mosso (Mosso, 1904). This proposition was met with considerable controversy and skepticism (Mosso, 1904; Tower, 1926; Cobb, 1918; Kanavel et al., 1925).

However, it was not until recently that satisfactory evidence was presented that the sympathetic nervous system modulates skeletal muscle activity at the NMJ in addition to the well-known innervation to the blood vessels that supply the muscle (Figure 1E; Solandt, 1942, Khan et al., 2016; Rodrigues et al., 2019). In 2016, Khan et al. established that sympathetic signaling at NMJs through β2-adrenergic receptors (β2AR) in mice is crucial for the integrity and stability of this neuron-muscle synapse (Figures 1F–K; Khan et al., 2016). Furthermore, sympathetic activation induces the import of peroxisome proliferator-activated receptor γ-coactivator 1α (PPARGC1A), which is crucial for mitochondrial biogenesis (Geng et al., 2010; Khan et al., 2016). β2AR agonism has also been reported to enhance synaptic vesicle exocytosis and acetylcholine release at the NMJs in the mouse diaphragm and to improve the contractile responses to phrenic nerve stimulation (Gafurova et al., 2024; Tsentsevitsky et al., 2020).

The sympathetic nervous system also regulates motor innervation and acetylcholine receptor (AChR) stability at NMJs (Rodrigues et al., 2019). A surgical lumbar sympathectomy involving the L2–L3 level sympathetic ganglia extensively alters the skeletal muscle transcriptome, affecting functions ranging from synaptic vesicle docking and fusion to circadian rhythm to motor denervation. This is correlated with alterations in NMJ transmission, evidenced by decreased muscle force generation in sympathectomized mice in response to short and prolonged motor nerve stimulation. A lag in sympathetic regeneration after peripheral nerve injuries may also underlie the inability of motor reinnervated skeletal muscle to recover from long-term functional deficits (Tian et al., 2025a).

Muscle weakness is a common symptom of diseases that involve the autonomic nervous system (Low et al., 2003; Delbono et al., 2021; Rodrigues et al., 2019; Khan et al., 2016; Pagani and Lucini, 1999, Stoica and Enulescu, 1992; Benarroch, 2024). Notably, sympathomimetics, such as the β2AR agonist salbutamol, have been effective at improving muscle strength exhibited in people with **congenital myasthenic syndromes** and animal models of **myasthenia**, which is defined as a condition that causes abnormal muscle weakness due to defective transmission at the NMJ (Stoica and Enulescu, 1992; Farmakidis et al., 2018; Alsallum et al., 2021; Milone and Engel, 1996; Guven et al., 2012; Lorenzoni et al., 2013). In 1992, Stoica and Enulescu found that patients with myasthenia exhibited an increase in epinephrine rather than norepinephrine after exercise, forearm ischemia, and orthostasis (Stoica and Enulescu, 1992). Because norepinephrine is the major neurotransmitter of postganglionic sympathetic neurons (Molinoff and Axelrod, 1971), this indicates a deficiency in the sympathetic nervous system response in myasthenic syndromes (Stoica and Enulescu, 1992). Not only does salbutamol improve physical symptoms in various mouse and zebrafish models of congenital myasthenic syndromes, it also increases the number of functional NMJs; synaptic area; AChR area, density, and clustering; and postjunctional folds (Webster et al., 2020; McMacken et al., 2018, 2019). Furthermore, salbutamol decreases errors in motor axon pathfinding exhibited in models of congenital myasthenic syndrome and restores impaired AChR **prepatterning** (McMacken et al., 2018), which is a process in which AChRs cluster and localize on muscle fibers in a central band prior to receiving motor innervation (Kummer et al., 2006). Therefore, sympathetic signaling at NMJs is important for NMJ formation, morphological integrity, and function.

**Sarcopenia**, which is the age-related decline in motor function, muscle mass, and strength (Frontera, 2022; Larsson et al., 2019; Lexell et al., 1988), has been linked to a decline in the feedback system between the skeletal muscle and muscle sympathetic nerves (Hotta et al., 2023, 2021). Resting sympathetic nerve activity in rats increases with age (Larkin et al., 1996), and this increase in sympathetic tone may be related to the muscle rigidity and pain experienced by older adults that is likely mediated by α-adrenergic receptors outside of NMJs (Hotta et al., 2023). Aged rats exhibit a smaller decrease in tetanic force following transection of the lumbar sympathetic trunk compared to young rats, indicating a decline in sympathetic modulation of tetanic force with age, and a larger increase in muscle tonus as a result of direct sympathetic stimulation independent of motor nerve stimulation (Hotta et al., 2023). The upregulation of *MuRF1*, which codes for a ubiquitin ligase that regulates ubiquitin-mediated degradation of skeletal muscle (Gumucio and Mendias, 2013), after sympathectomy leads to downregulation of postsynaptic AChR and muscle atrophy (Rodrigues et al., 2019). Additionally, administration of the β2AR agonist clenbuterol to rodents suppresses *MuRF-1* and *atrogin-1* gene expression in fast-twitch muscles in the context of motor denervation and fasting (Kline et al., 2007; Gonçalves et al., 2009). Similarly, isolated soleus muscles in rats who had undergone chemical or surgical sympathectomies exhibit decreased rates of protein synthesis that can be reversed with the addition of β2AR agonists (Navegantes et al., 2004). Furthermore, application of norepinephrine, the major neurotransmitter of postganglionic sympathetic neurons, on isolated extensor digitorum longus muscles from rats downregulates *MuRF-1* and *atrogin-1* expression as well as ubiquitin-proteasome activity (Silveira et al., 2014). Therefore, the sympathetic nervous system not only affects motor innervation of skeletal muscle, it also serves a protective role against muscle atrophy and sarcopenia (Gumucio and Mendias, 2013).

β2AR is the main receptor for skeletal muscle sympathetic signaling (Kim et al., 1991). β2AR signaling is linked to upregulation of PPARGC1A (Miura et al., 2007; Khan et al., 2016), and direct stimulation of the lumbar sympathetic chain induces a rapid increase in PPARGC1A in the myonuclei of mouse tibialis anterior muscles (Khan et al., 2016). Likewise, exercise also increases expression of *Ppargc1a* mRNA in mouse gastrocnemius muscles (Miura et al., 2007). This effect is ameliorated by blocking β-receptors and is recapitulated with agonism of β-receptors. Therefore, exercise-mediated upregulation of *Ppargc1a* is likely sympathetically-mediated through the activity of postsynaptic β2AR.

These findings illustrate the great importance of sympathetic signaling in skeletal muscle. Not only is the sympathetic nervous system critical for the stability of NMJs, it also plays roles in neurotransmitter release at the NMJ (Gafurova et al., 2024); muscle bulk, force, and tone (Stoica and Enulescu, 1992; Farmakidis et al., 2018; Alsallum et al., 2021; Milone and Engel, 1996; Guven et al., 2012; Hotta et al., 2023, 2021); and muscle mitochondrial biogenesis (Khan et al., 2016). Outside of its effects on the blood vessels supplying the muscle, the direct effect of sympathetic signaling on muscle through the NMJ is an area rich with old, new, and future discoveries.

## Zooming in: anatomy of sympathetic chain ganglia

The sympathetic nervous system is responsible for a wide array of bodily homeostatic mechanisms. The primary neurons in the sympathetic nervous system are the preganglionic sympathetic neurons located in the lateral horns of the thoracolumbar region (T1–L2) of the spinal cord and the postganglionic neurons located in the sympathetic chain ganglia, which span across all vertebra levels (McCorry, 2007). The resting and reflex regulation of the sympathetic nervous system is primarily governed by neurons in the **rostral ventrolateral medulla** (RVLM), which is located in the brainstem (Kulkarni et al., 2023; Kumagai et al., 2012). The RVLM is critical for the continuous regulation of the tonic sympathetic drive, which controls resting blood pressure and can differentially regulate sympathetic activity to each organ based on physiological demands, indicating functional separation (Guyenet, 2006; Schreihofer and Sved, 2011; Mcallen et al., 1995; Mueller et al., 2011).

In humans, the sympathetic chain ganglia, or paravertebral ganglia, that house the **postganglionic sympathetic neurons** are located on either side of the spinal column, retroperitoneally, and extend from the base of the skull to the coccyx with cervical, thoracic, lumbar, and sacral segments (Jetti et al., 2019). Postganglionic sympathetic neurons, which are unmyelinated, send their projections through the **gray rami communicans** to join the spinal nerves to reach their peripheral targets. There are 3 cervical sympathetic ganglia: superior, middle, and inferior. The **stellate ganglion**, also known as the cervicothoracic ganglion, is the fusion of the inferior cervical ganglion and the first thoracic ganglion (Jamieson et al., 1952), predominantly located in the first intercostal space and is found in about 75%−82% of thoracic sympathetic trunks (Vanlommel et al., 2022; Street et al., 2016; Pather et al., 2006; Jamieson et al., 1952). In the thoracic region, the upper thoracic ganglia are mostly located in their respective intercostal spaces, and the lower thoracic ganglia exhibit a downward shift (Vanlommel et al., 2022).

The lumbar sympathetic chain lies anterolateral to the lumbar vertebrae bodies and is continuous with the thoracic sympathetic chain superiorly (Jetti et al., 2019). Inferiorly, the lumbar sympathetic chain lies behind the common iliac vessels and is continuous with the sacral sympathetic chain. The right chain lies posterior to the inferior vena cava, and the left chain lies posterolateral to the aortic lymph nodes. The positions of the L1–L4 lumbar sympathetic ganglia have been found to exhibit variability in respect to their positions relative to the vertebral body, with the L5 ganglion being the most consistent in location (Lowenberg and Morton, 1951; Jain et al., 2016). In 1951, Lowenberg and Morton performed an anatomical study of 58 human lumbar sympathetic chain ganglia and found that the number of discrete ganglia ranged from 1 to 5, with 4 being the most common (Lowenberg and Morton, 1951). There were many intercommunications between the left and right lumbar chains, which may render bilateral sympathectomies desirable in cases of severe lower extremity vasospastic disease to remove any contralateral impulses.

**Figure 2:**
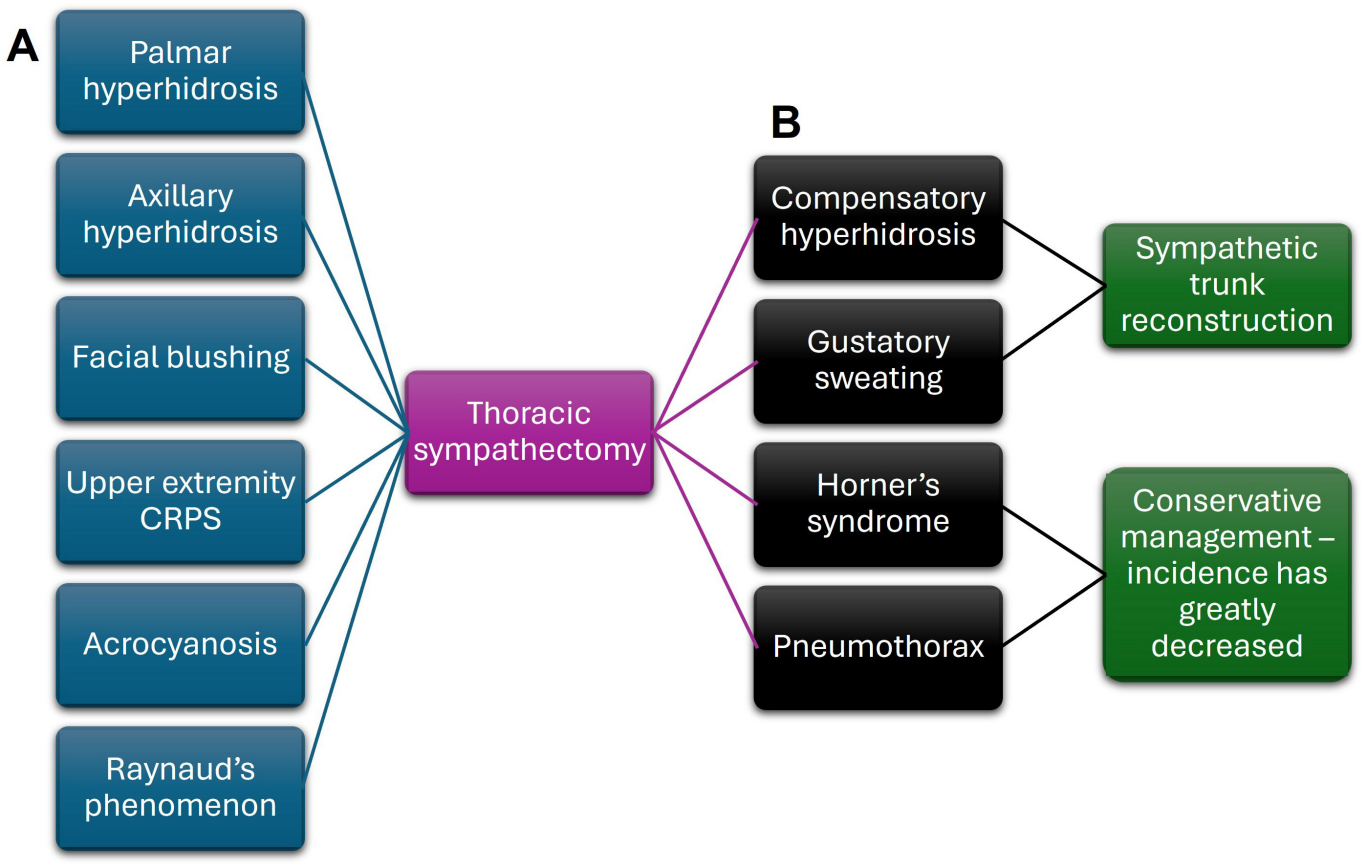
Clinical relevance of thoracic sympathectomy. **(A)** A thoracic sympathectomy has been applied to many clinical scenarios. **(B)** Side of effects of this surgical intervention can be either managed by sympathetic trunk reconstruction or by conservative management.

Understanding the anatomy of the paravertebral ganglia, their variations, and their interconnectedness has paved the way for surgical sympathectomies that can treat a variety of disease states such as hyperhidrosis (Dumont et al., 2004), complex regional pain syndrome (Schwartzman et al., 1997; Singh et al., 2003; Alkosha and Elkiran, 2016), vasospastic diseases such as acrocyanosis and Raynaud's phenomenon (Figure 2A; Di Lorenzo et al., 1998), and more (Licht et al., 2006; Ouriel and Moss, 1995). However, the identification of the lumbar sympathetic chain in human sympathectomies can be difficult due to the required depth of the surgical exposure, the proximity of neighboring vital structures, and the similarity in appearance of the sympathetic chain to the genitofemoral nerve and lymphatic system, which can result in off-target surgical excisions (Lowenberg and Morton, 1951).

In mice, the thoracic sympathetic ganglia are located in the intercostal spaces within their respective vertebra level, which is similar to in humans. The location of the lumbar sympathetic ganglia in mice, however, is slightly differently than in humans though similar to the anatomical location in rats. The L2–L5 level lumbar sympathetic ganglia traverse from the level of the left renal artery to the level of the bifurcation of the aorta into the iliac arteries and are most easily accessed through a transabdominal approach (Tian and Ward, 2024; Zhao et al., 2007; Zheng et al., 2017). The bilateral chains are situated in the animal's midline, behind the abdominal aorta and inferior vena cava, between the bilateral psoas muscles. The L1 level ganglia can be located on either side of the diaphragmatic crura (Tian et al., 2025b; Tian and Ward, 2024).

Reliable identification of the lumbar sympathetic ganglia can allow for the surgical removal of this group of neurons in animal models to better understand how peripheral targets in the lower body change without sympathetic input. The ability to do this in mice paves the way for the combination of genetic manipulations and surgical interventions that can only be achieved in mouse models (Tian and Ward, 2024).

## Clinical side effects of surgical sympathectomies

The use of surgical sympathectomies has been explored in numerous disease states that involve sympathetic overactivity with varying results. However, surgical sympathectomies can come with unwanted side effects and complications. Some even postulate that the evidence supporting the use of surgical sympathectomies is poor, mainly relying on uncontrolled studies and personal experience (Mailis and Furlan, 2003). Adverse effects and complications include worsening pain or new pain as well as abnormal forms of sweating, such as compensatory hyperhidrosis and pathological gustatory sweating, Horner's syndrome, and pneumothorax (Figure 2B; Mailis and Furlan, 2003; Dumont et al., 2004; Licht and Pilegaard, 2004). The incidence of Horner's syndrome, which often occurs due to excessive manipulation and cautery on the sympathetic chain, has greatly decreased thanks to refinements in operative techniques and appreciation of the surgical anatomy, such as avoiding the cervicothoracic ganglion (Vanlommel et al., 2022; Street et al., 2016; Pather et al., 2006; Jamieson et al., 1952; Singh et al., 2006). Although pneumothorax often resolves with conservative management, it can occur in up to 25% of patients (Licht and Pilegaard, 2004). Due to the high incidence of adverse effects, patient regret ranges from 13.5% to 16% for thoracic sympathectomies performed to treat primary hyperhidrosis and facial blushing (Smidfelt and Drott, 2011; Licht and Pilegaard, 2004).

The most common indication for the use of sympathectomies is palmar hyperhidrosis, which is achieved via thoracoscopic ablation of the T2–T4 thoracic ganglia, though specific levels within this range varies (Gordon et al., 1994; Smidfelt and Drott, 2011; Weksler et al., 2008; Chang et al., 2007). In a study in 2011 that included 1700 patients who underwent bilateral thoracic sympathectomies, the use of endoscopic thoracic sympathectomies for palmar hyperhidrosis resulted in relatively high patient satisfaction, with an 86.6% satisfaction rate accompanied by a 95.6% efficacy rate (Smidfelt and Drott, 2011). This study also found that, in contrast, patients with axillary hyperhidrosis, facial hyperhidrosis, and facial blushing were less satisfied after bilateral thoracic sympathectomy, with 66.0%, 74.0%, and 73.5% satisfaction rates, respectively. Notably, **compensatory hyperhidrosis** was present in 80% of the study participants. In a separate study in 2004 that included 89 patients, compensatory sweating occurred in 87% of the study participants who underwent bilateral T2–T4 sympathectomies, reaffirming the pervasiveness of this particular side effect (Dumont et al., 2004). Compensatory hyperhidrosis is called “compensatory” because it was believed to be a thermoregulatory response to the new onset anhidrosis induced by the sympathectomy (Shelley and Florence, 1960). Even through sweating is decreased or eliminated in the original area of interest, sweat production is increased in other locations, most commonly the abdomen, back, legs, and gluteal area, which fall outside of the dermatomal range of the sympathetic ablation (De Campos et al., 2003, Ojimba and Cameron, 2004). Therefore, abnormal sympathetic sprouting may also be a component of this phenomenon, in which intact sympathetic fibers become sensitized while also trying to reinnervate denervated areas (Gloster and Diamond, 1992; Murray and Thompson, 1957; Kinnman, 1986; Ahčan et al., 1998). This is supported by the fact that more extensive thoracic ganglionectomy can be utilized to remedy compensatory sweating, though notably, the maximum follow-up time for any of the 8 patients included in this particular study was 26 months (Yamamoto and Okada, 2019).

**Gustatory sweating**, which is facial sweating when eating particular foods, is also a noted side effect of thoracic sympathectomies for treatment of hyperhidrosis (Licht and Pilegaard, 2004; Mailis and Furlan, 2003). In 2004, a cohort of 158 patients were evaluated for differences in compensatory sweating and gustatory sweating after thoracic sympathectomies at different levels (T2 for facial hyperhidrosis/blushing, T2–3 for palmar hyperhidrosis, and T2–4 for axillary hyperhidrosis; Licht and Pilegaard, 2004). Compensatory hyperhidrosis was reported by 89% of patients, further reiterating that this side effect is extremely common, with 35% of patients reporting that the sweating was so severe that they had to change their clothes during the day. Greater severity of compensatory sweating was associated with the more extensive T2–T4 sympathectomies, which appears to contradict the conclusions from Yamamoto and Okada's study mentioned previously, emphasizing that performing extensive ganglionectomies is still controversial (Licht and Pilegaard, 2004; Yamamoto and Okada, 2019). Gustatory sweating was reported by 38% of participants and was associated with the consumption of spicy foods and foods with moderate acidity such as apples and oranges (Licht and Pilegaard, 2004). Although the underlying cause of gustatory sweating is unclear, vagal nerve sprouting into the severed sympathetic chain has been implicated alongside collateral sprouting of residual sympathetic fibers (Hashmonai et al., 1992; Bloor, 1969; Shoenfeld et al., 1976; Murray and Thompson, 1957). This phenomenon is unlikely to be caused by hypersensitivity of denervated sweat glands to acetylcholine is since human sweat glands actually exhibit decreased sensitivity following denervation (Kahn and Rothman, 1942; Janowitz and Grossman, 1950, 1951); however, sweat glands in the areas affected by gustatory sweating do exhibit hypersensitivity, suggesting that reinnervation with parasympathetic involvement affects receptor sensitivity, though this theory is not well-supported (Iwabuchi et al., 1966). A new connection between preganglionic vagal nerve fibers and preganglionic sympathetic fibers that innervate the sweat glands has been posited, with efferent vagal nerve impulses driving sweat gland activation since both of these preganglionic fibers use acetylcholine as their primary neurotransmitter. Frey's syndrome, a rare complication following parotid or submaxillary gland surgeries, trauma, or radical neck dissections, manifests similarly, with localized gustatory sweating specifically in the preauricular area (Gordon and Fiddlan, 1976; Drummond et al., 1987). Similar to gustatory sweating post-sympathectomy, aberrant parasympathetic reinnervation of sweat glands is likely involved in the manifestation of Frey's syndrome, but this mechanism is more likely to be through aberrant parasympathetic invasion into previously sympathetically-innervated areas after injury of the auriculotemporal nerve (Drummond, 2002; Dunbar et al., 2002).

With the severity of some of the side effects experienced by patients who undergo sympathectomies, which can be greatly debilitating and affect their quality of life, reversal of surgical sympathectomies has been explored since the late 1990s (Telaranta, 1998). Telaranta published one of the first cases of sympathetic chain reconstruction following electrocautery-based thoracic sympathicotomy, originally perceived to be an irreversible procedure, via open nerve reconstruction of the transected sympathetic chains in 1998. The patient reported relief from compensatory hyperhidrosis and return of facial and armpit sweating 1 year after the reconstruction. Rantanen and Telaranta went on to report that sympathetic trunk reconstruction with intercostal or sural nerve grafts improved compensatory sweating in 15 of their 19-patient cohort in 2017 (Rantanen and Telaranta, 2017). From 2004 to 2007, a group at Yonsei University College of Medicine performed 19 sympathetic chain reconstructions using intercostal nerve grafts with 9 patients reporting a reduction in their compensatory hyperhidrosis symptoms (Haam et al., 2010).

In the last 5 years, reversal of thoracic sympathectomies via robotic-assisted sympathetic trunk reconstruction has been galvanized by an interdisciplinary group at Chang Gung Memorial Hospital (Chang et al., 2021, 2020; Chen et al., 2023). In 2020, Chang et al. published a case series describing the feasibility of using a sural nerve graft for reconstruction of the thoracic sympathetic chain (Chang et al., 2020). In a prospective study of 23 patients, the severity of compensatory sweating was significantly reduced at 6 months post-reconstruction, and this improvement was sustained 2 years after surgery (Chen et al., 2023). As of January 2025, this interdisciplinary team has performed 161 sympathetic trunk reconstructions, which includes both thoracic and lumbar regions, and has also transitioned to use intercostal nerve grafts due to the proximity of these donor nerves to the thoracic sympathetic trunk (1 February 2025, International Microsurgery Club Webinar).

## Discussion

Novel insights into the sympathetic nervous system have led to renewed scientific interest in the role of sympathetic innervation of peripheral tissues and its contribution to various neurological and neurodegenerative diseases. Sympathetic dysregulation can be detected by an abnormal SSR, which is associated with several neurological and neurodegenerative diseases. Although changes in SSR are often detected before primary presentations of certain neurological conditions, the diagnostic utility of SSR still remains limited. The absence of the SSR has been found to reliably indicate the presence of autonomic neuropathy in patients with diabetes, with a sensitivity of 87.5%, a specificity of 88.2%, and a negative predictive value of 93.7% (Al-Moallem et al., 2008). However, there have not been any specific symptoms identified to be correlated with autonomic involvement in diabetic neuropathy (Soliven et al., 1987). The SSR has also been studied as a potential predictor of diabetic cardiac autonomic neuropathy, but even after establishing optimal cutoff points for SSR amplitudes, the sensitivity and specificity ranged from 61.9% to 68.5% (Lin et al., 2021). Despite the limited evidence to support the diagnostic utility of the SSR in diabetic neuropathy, there is a great need for future studies to expound upon the SSR changes in a multitude of neurological conditions, therefore harnessing the potential of SSR testing in early disease detection and predicting disease progression.

Within the skeletal muscle, outside of the typical vascular regulatory response associated with the sympathetic nervous system, sympathetic signaling in skeletal muscle is important for the normal development of NMJs, maintaining NMJ integrity, modulating neurotransmitter release, and regulating skeletal muscle tone. Additionally, newfound appreciation of sympathetic dysregulation has prompted new studies into the role of sympathetic signaling in sarcopenia. Correcting the underlying sympathetic dysregulation opens up several avenues of future exploration that could become the basis of future therapeutics for neurological disorders and age-related functional decline.

Sympathetic dysregulation after surgical ablation, a procedure initially developed for the treatment of heightened sympathetic activity, has prompted the development of reconstructive surgeries that seek to reverse the adverse side effects associated with the removal of parts of the sympathetic chain ganglia. These adverse side effects illustrate the importance of an intact sympathetic nervous system for regulating bodily homeostasis. Although local approaches for hyperhidrosis are available, such as botulinum toxin injections, topical medications, and iontophoresis, they do not yet offer a permanent solution (Walling and Swick, 2011). Therefore, more targeted, permanent approaches, that do not involve injury to the sympathetic chain, should be explored. Because sympathetic activity of the skin and muscle are independent from one another (Middlekauff et al., 1994), investigations into the development of therapeutics that target sympathetic skin activity and not sympathetic muscle activity should be undertaken for the treatment of conditions such as palmar hyperhidrosis. By deepening our understanding of the intricacies of the sympathetic nervous system and its myriad of functions, we position the field for discoveries that may change our approach to neuroscience research and clinical practice.
